# Effects of stimuli and contralateral noise levels on auditory cortical potentials recorded in school-age children

**DOI:** 10.1371/journal.pone.0317661

**Published:** 2025-01-22

**Authors:** Thalita Ubiali, Camila Colussi Madruga-Rimoli, Thais Antonelli Diniz-Hein, Milaine Dominici Sanfins, Bruno Sanches Masiero, Maria Francisca Colella-Santos

**Affiliations:** 1 Faculdade de Ciências Médicas, Universidade Estadual de Campinas (UNICAMP), Campinas, São Paulo, Brazil; 2 Department of Speech-Hearing-Language, Universidade Federal de São Paulo, São Paulo, Brazil; 3 Instituto de Ensino e Pesquisa Albert Einstein, Post-Graduate Program in Clinical Audiology, São Paulo, Brazil; 4 Department of Teleaudiology and Screening, Institute of Physiology and Pathology of Hearing, World Hearing Center, Kajetany, Poland; 5 Faculdade de Engenharia Elétrica e de Computação, Universidade Estadual de Campinas (UNICAMP), Campinas, São Paulo, Brazil; Universiti Kebangsaan Malaysia, MALAYSIA

## Abstract

**Background and objective:**

One of the functions attributed to the auditory efferent system is related to the processing of acoustic stimuli in noise backgrounds. However, clinical implications and the neurophysiological mechanisms of this system are not yet understood, especially on higher regions of the central nervous system. Only a few researchers studied the effects of noise on cortical auditory evoked potentials (CAEP), but the lack of studies in this area and the contradictory results, especially in children, point to the need to investigate different protocols and parameters that could allow the study of top-down activity in humans. For this reason, the aim of this study was to analyze the effect of varying levels of contralateral noise on efferent activity in children by recording CAEPs with tone burst stimuli. Additionally, we aimed at verifying the effects of contralateral noise on cortical processing of speech stimuli.

**Methods:**

Monaural CAEPs were recorded using tone burst stimuli in quiet and with contralateral white noise at 60 dB and at 70 dB in 65 typically developing school-aged children (experiment 1), and using speech stimuli with contralateral white noise at 60 dB in 41 children (experiment 2).

**Results:**

In experiment 1, noise induced changes were observed only for P1 and P300 components. P1 latency was prolonged at both noise level conditions, P300 latency was prolonged only in the condition with noise at 70 dB, and P300 amplitude was reduced only in the condition with noise at 60 dB. In experiment 2, noise induced latency delays were observed on P1, P2, N2, and P300 components and amplitude reduction was observed only for N1.

**Conclusion:**

The effects of noise stimulation were observed on all CAEP components elicited by speech, but the same was not observed in the experiment with tone bursts.

The study of noise effects on CAEPs can provide electrophysiological evidence on how difficult listening situations affect sound discrimination and stimulus evaluation at thalamocortical regions.

## Introduction

Human hearing is a complex and sophisticated process that makes it possible to focus on sounds of interest while ignoring irrelevant background noise. This ability is related to the adaptive feature of the auditory system, which can modulate its activity to extract predictable/repetitive information of someone’s voice (e.g., pitch, duration, and intensity patterns) from random competing noise [1,2]. Several factors can contribute to this process including sensory encoding in the peripheral and central auditory systems [3], as well as top-down/cognitive abilities (such as attention, short-term memory and language) [2]. It is known that top-down processing (via corticofugal pathways) can influence tuning of sensory information at auditory periphery and central subcortical regions, enhancing features of the target speech signals (e.g., the fundamental frequency), and facilitating speech perception in noise [2,4]. Several researchers have investigated the role of the auditory efferent system in speech-in-noise processing [5–7] and although it is known that auditory cortical areas can modulate peripheral activity through the cortico-olivocochlear pathway [5–15], the anatomo-physiological mechanisms underlying efferent activation at higher regions of the central auditory nervous system (CANS), especially in noisy backgrounds, remain unknown.

Recently, researchers are gaining increasing interest in the use of cortical auditory evoked potentials (CAEPs) to investigate the effects of competing noise on cortical encoding of acoustic signals, in part, because they provide a non-invasive technique for studying sensory and cognitive processing of sounds in humans [16–24]. In general, CAEP components tend to present longer latencies and smaller amplitudes when competitive noise is added to the testing [25]. However, previous studies demonstrated divergent results when comparing CAEP responses in quiet and noise conditions. In adult populations, both reduction and enhancement of P2 elicited by tones were reported in the presence of noise stimulation [18,19]. Another study [22] described enhanced N2 amplitude in noise when the elicitor stimuli were speech, but no significant changes occurred for complex tones. Studies with school-aged children showed no changes on P2-N2 amplitude in the presence of binaural competing noise, but the authors found reduced amplitude with ipsilateral noise and increased amplitude with contralateral noise [24]. Regarding P300, some studies demonstrated increased latency in the presence of noise [16,17,23] while others reported no significant changes [19,20,22] in adults. Those conflicting findings might be due to different collecting and analysis parameters, such as elicitor stimulus, signal-to-noise ratio (SNR), passive or active listening condition, task complexity, and methods of analysis. In addition, studies about the effects of noise on P300 with the pediatric population are rather scarce. Ubiali et al. [21] reported noise-induced changes on P300 latency, but no changes in amplitude for CAEPs elicited with tone bursts. To the best of our knowledge, no other study has performed such an investigation of noise effects on the cognitive potential P300 in school-aged children.

The lack of studies in this area, especially in the pediatric population, points out the need for more research using CAEPs in noise conditions and investigating different parameters of acquisition. For this reason, the purpose of the present research was to study different parameters (such as contralateral noise levels and stimulus type) for assessing the effects of efferent activity on the cortical auditory processing of children. Therefore, the study was divided into two experiments. The first experiment was designed to compare the 60 dB SPL and 70 dB SPL contralateral noise on CAEPs obtained with tone bursts. Consistent with previous work [3,21], we hypothesized that the 70 dB noise level would induce greater effects of noise on CAEPs (e.g., longer latencies and reduced amplitudes) because it represents a more difficult listening condition. In the second experiment, we aimed at verifying if competing noise would similarly influence CAEP responses obtained with speech and nonspeech stimuli. Given that speech stimuli are more complex than tones, our hypothesis was that CAEPs elicited by speech would be more degraded by noise than the ones elicited by tone bursts [25].

## Methods

### Ethics statement

This study was approved by the Ethics in Research Committee of the State University of Campinas, Campinas, São Paulo, Brazil, under the protocol number 2.116.400. Data was collected by the author from August 2017 to August 2019 at the Laboratory of Audiology of the Department of Human Development and Rehabilitation, Faculty of Medical Sciences, UNICAMP. Written informed assent and consent were obtained from children and their parents/legal guardians to participate in the study.

### Participants

Sixty-five typically developing children (38 females) aged between 8 and 13 years (mean = 10.1, SD = 1.4) participated in experiment 1. Forty-one of these children (21 females) between 8 and 13 years of age (mean = 10.6, SD = 1.4) also participated in experiment 2. Table 1 shows the number of participants by age and gender. Right-handed and normal hearing children (defined as pure-tone thresholds ≤ 15 dB at 250 to 8000 Hz; normal speech recognition index [26]; type A tympanometry and the presence of ipsi and contralateral acoustic reflexes [27]) were recruited from public elementary schools. Other inclusion criteria were peak latencies of waves I, III, and V for click-evoked Auditory Brainstem Response (ABR) within clinically normal ranges (SmartEp, Intelligent Hearing System) and good academic performance (according to a questionnaire completed by their classroom teachers). Children’s medical and developmental history was informed by their parents. Exclusion criteria were: hearing loss, history of recurrent otitis media, poor academic performance, suspected or diagnosed learning difficulties, language problems, attention deficits or other neurological or psychiatric conditions, and current use of psychoactive drugs.

**Table 1:** Number of participants by age and gender.

	Experiment 1			Experiment 2		
Age	Male (n)	Female (n)	Total (n)	Male (n)	Female (n)	Total (n)
8 years	5	8	13	1	5	6
9 years	2	7	9	1	4	5
10 years	2	10	12	3	2	5
11 years	7	5	12	5	4	10
12 years	9	7	16	7	5	12
13 years	2	1	3	2	1	3
Total (n)	27	38	65	19	21	41

### Procedures

Participants were submitted to the following procedures: otoscopy, pure tone audiometry, tympanometry, ABR, and CAEPs assessment with tone burst stimuli in three conditions: 1) quiet (Q), 2) with contralateral noise at 60 dB, and 3) with contralateral noise at 70 dB. These procedures were performed in a session lasting approximately two hours (experiment 1). Participants were invited to attend a second session to perform CAEPs recordings with speech stimuli in quiet and 60 dB contralateral noise conditions (experiment 2). Forty-one participants agreed to return in the second session. The two sessions were taken approximately one to two weeks apart.

CAEP testing was performed in a sound-attenuated and electrically-shielded room where subjects were comfortably accommodated in a cushioned recliner. The equipment used was a 2-channel SmartEP, Intelligent Hearing System, and surface electrodes were placed on participants’ head on the following positions: active electrode on the vertex (Cz), the reference electrodes on the right (M2) and left (M1) mastoids and the ground on the forehead (Fz) (10–20 system) [28]. Impedance values were maintained at below 5 kΩ. Responses were bandpass filtered from 1 to 30 Hz and amplified with a gain of 50 K at a sampling rate of 1000 Hz. Recording time window was 513 ms.

CAEPs were elicited through an oddball paradigm with target stimuli randomly presented at a probability of 20% and standard at 80% probability.

In **experiment 1**, tone burst stimuli were used to elicit CAEPs. Target stimulus was 2KHz (rise / fall: 20 ms, duration: 100 ms) and the standard was 1kHz (rise / fall: 10 ms, duration: 50 ms), both presented at 70 dB nHL. Following the evaluation in quiet, recording was repeated with continuous white noise presented to the contralateral ear. Two noise levels were tested: 60 dB SPL and 70 dB SPL. The order of noise presentation (60 dB and 70 dB) was randomized across subjects. The decision of using the 2KHz and 1KHz (oddball paradigm) elicitors, as well as the contralateral competing white noise, was based on our previous work that reported the effects of competing noise (at 70 dB SPL) on P300 in school-age children [21]. Considering that lower white noise intensities are preferable with the purpose of minimizing the activation of middle ear muscle reflex (MEMR) [14], in the present study, we sought to verify if a lower noise level such as 60 dB would produce the same expected effect (delayed latency) on P300 as previously reported.

In **experiment 2**, CAEP was recorded with speech stimulation in the absence and presence of continuous contralateral white noise. Target stimulus was the syllable /da/ (F0: 109.1–102.1 Hz, F1: 732 Hz, F2: 1335 Hz, F3: 2498 Hz, F4: 3058 Hz, F5: 3828 Hz) and the standard stimulus was the syllable /ba/ (F0: 112.4–111.2 Hz, F1: 818 Hz, F2: 1378 Hz, F3: 2024 Hz, F4: 2800 Hz, F5: 4436 Hz), provided by SmartEP/Intelligent Hearing System. Elicitor stimuli were both presented at 70 dB nHL. For the purpose of optimizing time, contralateral white noise was presented only at 60 dB, because the objective here was to verify the effect of stimuli rather than competing noise levels.

Stimuli elicitors were monaurally presented to the right and left ears through insert earphones (ER-3A; Natus Medical) at a presentation rate of 1.1 stimuli per second, in a total of 300 sweeps in each condition. The order of the stimulated ear was alternated between subjects, that is, if a subject was first tested in the right ear, the next participant was first tested in the left. Children were instructed to mentally count the target stimuli and lift their index finger whenever they heard it. A brief training was carried out prior to evaluation with the purpose of ensuring adequate understanding of the task, that is, children should correctly identify at least four out of five target stimuli before proceeding to testing. At the end of the evaluation, participants were asked how many targets they counted, so that the examiner was able to ensure they performed the task properly. Participants who did not perform the task properly (e.g., who guessed the number of targets) were not included in the study. Children were also told to keep their eyes closed during recordings to avoid eye movement interference. Whenever necessary, children were given a few minutes break to avoid for fatigue. Electrode set up remained positioned on the child during breaks.

### CAEP analysis

**Fig 1:**
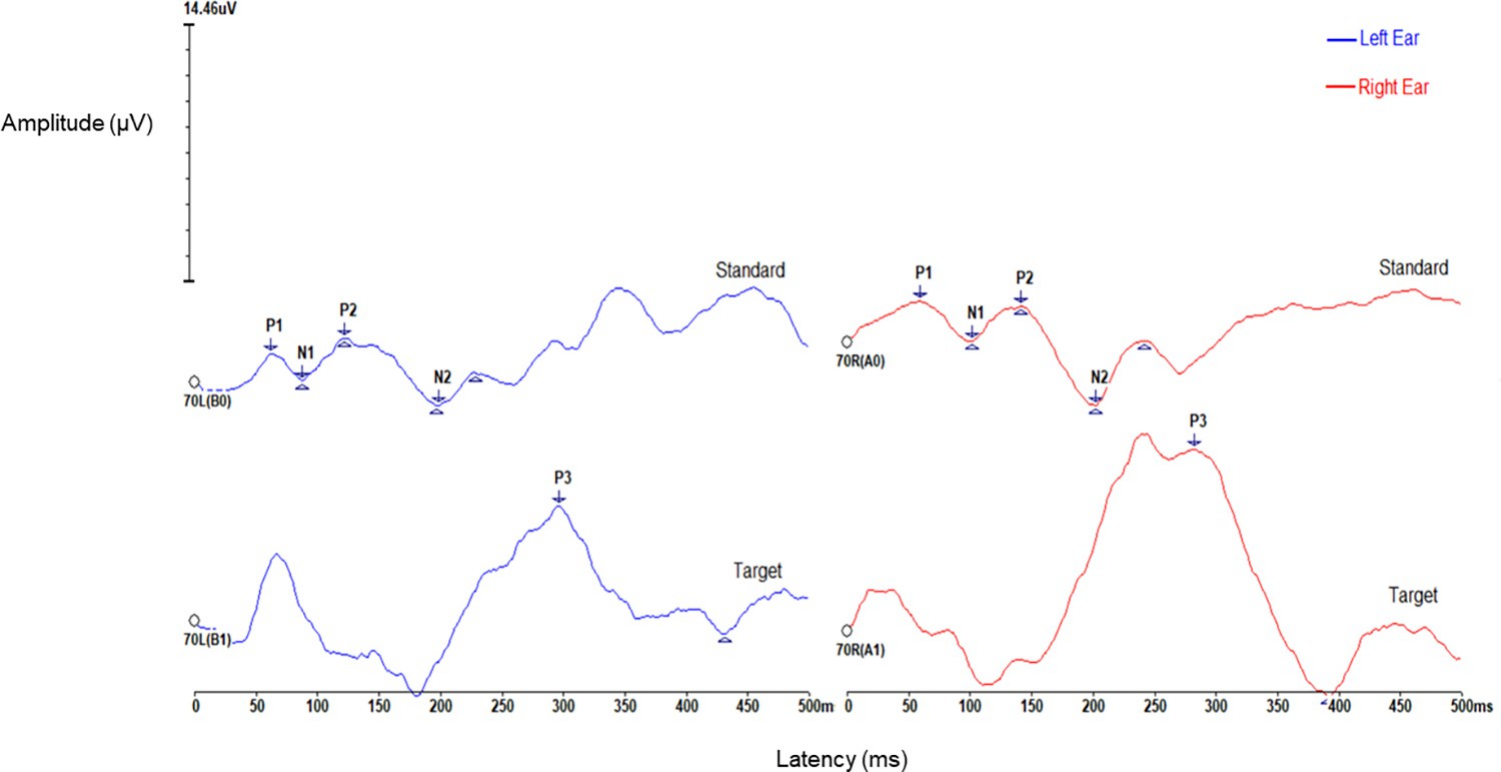
Example of CAEP analysis. P1-N1-P2-N2 complex was marked on the trace corresponding to standard stimuli and P300 (P3b) was marked on the trace corresponding to target stimuli. Waveforms were obtained from one subject of the sample (female, 10 years old). Blue waveforms correspond to the left year and red waveforms to the right ear.

Analysis was based on the values of latency (ms) and amplitude (μV) in the conditions quiet and with contralateral noise. Amplitude and latency values for P1, N1, P2, N2, and P300 peaks were obtained separately for each component. P1 was identified at the most positive peak prior to N1. The N1 peak was defined as the first most negative deflection, followed by a positive peak defined as P2. N2 was established at the most negative deflection after P2. The P1-N1-P2-N2 complex was analyzed in the trace corresponding to the standard (frequent) stimulus. P300 was analyzed at the most positive peak on the responses to the targets (rare) waveform, between 220 and 390 ms [29]. Whenever there was a double peak, P300 was marked at the second peak (P3b). Peak amplitude was measured from the most positive peak to the next most negative valley (P1, P2 and P300) and from the most negative valley to the next positive peak (N1 and N2). Fig 1 displays two examples of CAEP analysis from one subject of our sample. Waveform analysis was performed by two researchers, one of them blinded to the assessment condition (quiet, noise 60 dB, and noise 70 dB) and there should be agreement between them. Fifteen waveforms required a third (blinded) researcher consultation and there should be a consensus between two of them. Immature waveform morphology (i.e., P1-N2 peaks instead of P1-N1-P2-N2) was observed in 22 participants in experiment 1 and 16 participants in experiment 2. Recordings with artifact values greater than 10% were repeated to obtain a reliable response with fewer artifacts. Recordings that remained with a high level of interference were excluded from analysis: this resulted in the exclusion of four participants in experiment 1 and the exclusion of one participant in experiment 2. We omitted any participants with missing data for any Noise Condition or Ear for the current component, that is, only paired values were available for each dependent variable. The final sample for each component was P1 = 49, N1 = 45, P2 = 46, N2 = 59, and P300 = 61 participants for tone burst stimuli (experiment 1) and P1 = 33, N1 = 24, P2 = 24, N2 = 34, and P300 = 40 participants for speech stimuli (experiment 2).

### Statistical analysis

Statistical Analyses were conducted in R (Version 3.6.1, R-project: https://www.r-project.org), using the gamlss function from the gamlss package. Data normality and homogeneity of variances were observed from graphic inspection of the residuals. We analyzed amplitude and latency separately for each CAEP component with a linear mixed effects regression model. The model included a random effect of subjects (to account for individual differences) and the fixed effect of noise conditions (quiet, noise 60dB, and noise 70dB in experiment 1; and quiet and noise 60dB in experiment 2). To find out whether ear (right and left), sex (male and female) and age (8, 9, 10, 11, 12, and 13 years old) had any influence on the results, these variables were also included as fixed effects in the model. In experiment 2 we also used the Kruskall-Wallis test to test for stimuli differences (tone x speech) for the means of latency and amplitude of each CAEP in each noise condition. Confidence intervals were established at 95% and significance level was set at 5% (p<0.05). Parameter estimate tables can be found in the Appendixes and the statistically significant values were highlighted in bold (p < 0.05).

## Results

### Experiment 1

**Fig 2:**
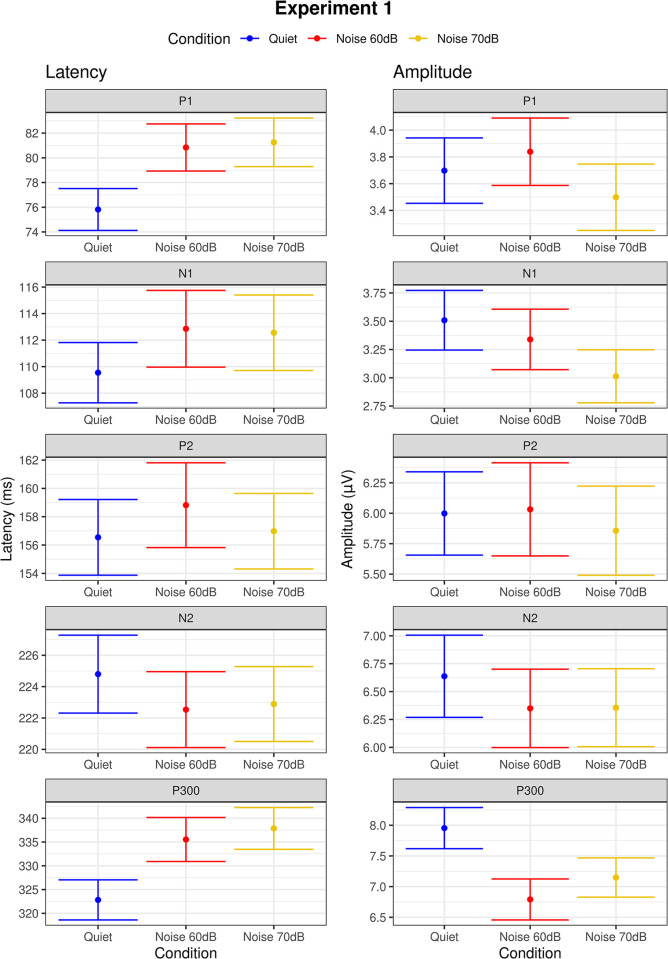
Error bars showing the means and standard errors for the latencies (ms) and amplitudes (μV) of CAEPs obtained with tone burst stimuli in the conditions quiet (in blue), noise 60 dB (in red), and noise 70 dB (in yellow). Dots indicate the mean and error bars indicate the margin of 1 standard deviation around the mean.

**Fig 3:**
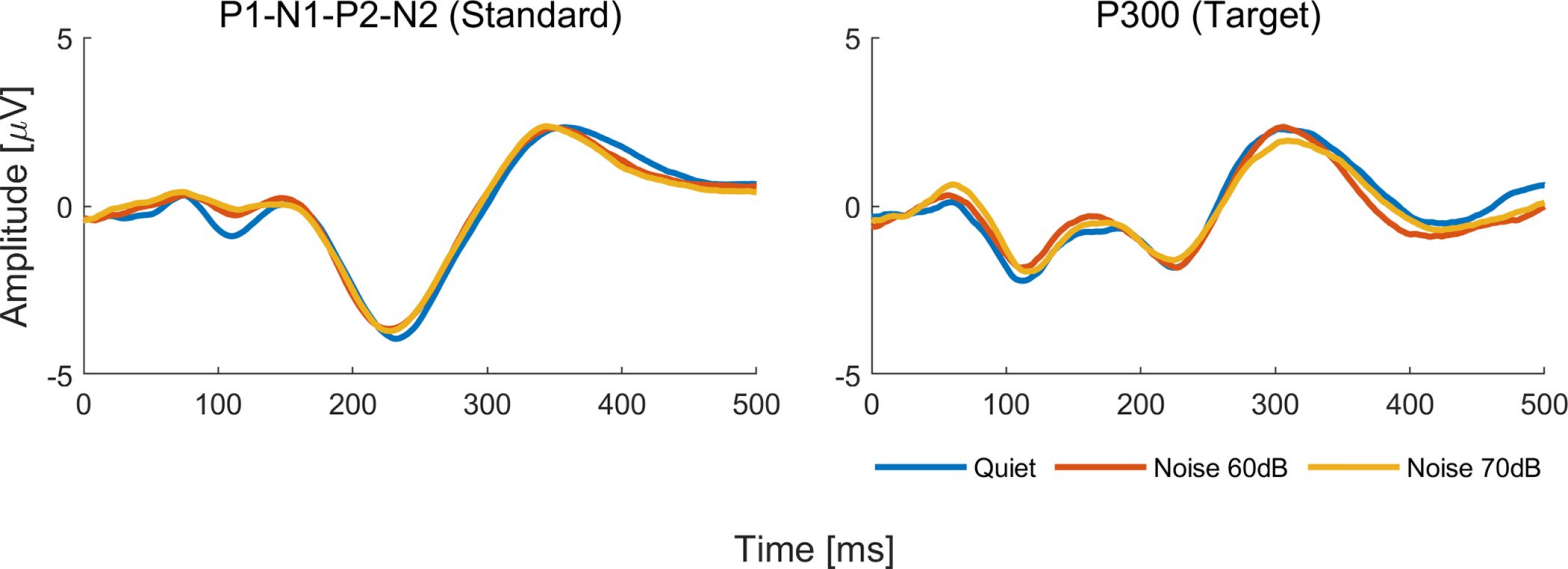
Grand average waveforms for responses to tone burst stimuli in quiet (blue line), in competing contralateral noise at 60 dB (red line), and in competing contralateral noise at 70 dB (yellow line).

**Fig 4:**
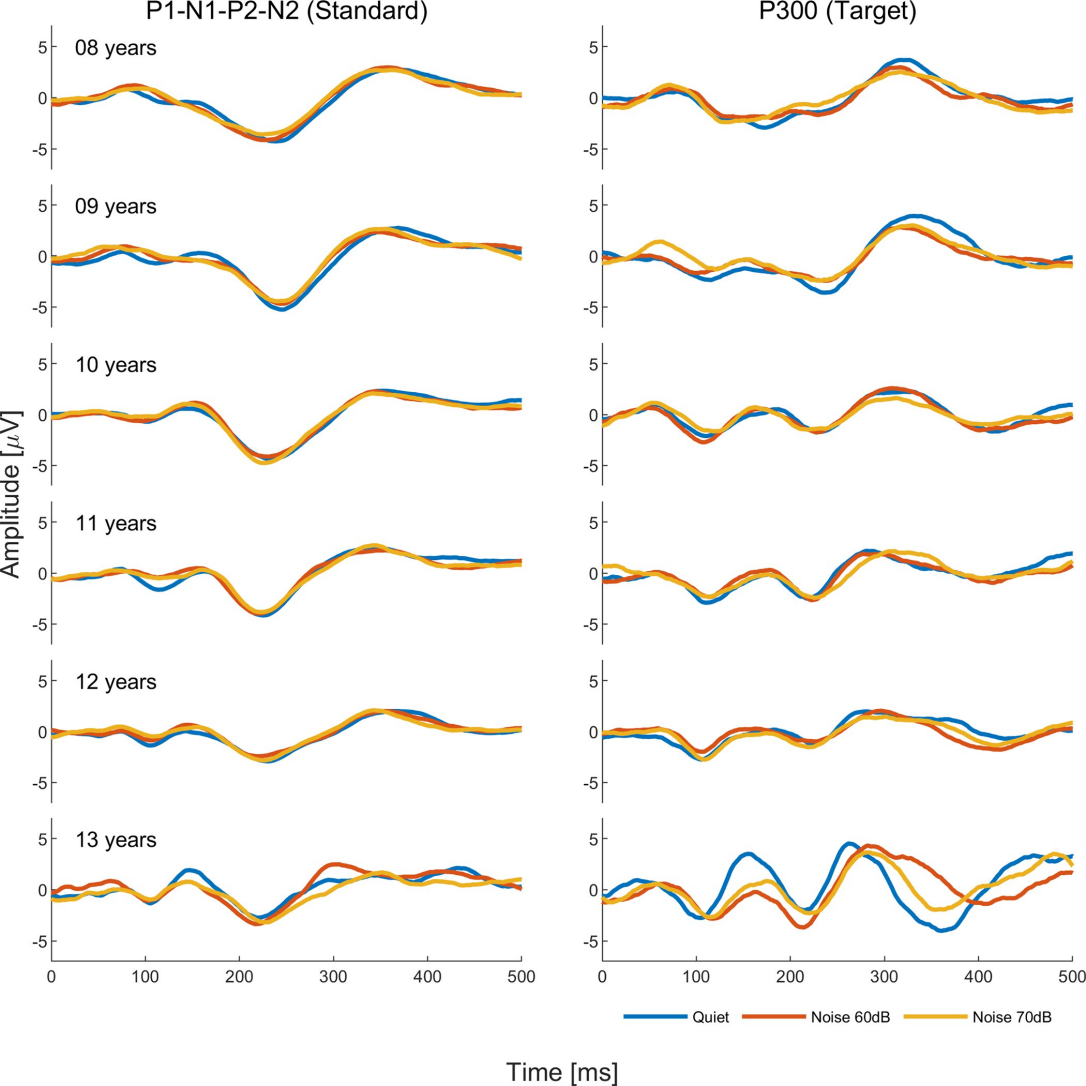
Grand average waveforms by age for responses to tone burst stimuli in quiet (blue line), in competing contralateral noise at 60 dB (red line), and in competing contralateral noise at 70 dB (yellow line).

Model analysis for tone burst CAEPs showed differences between quiet and noise conditions for P1 latency (*p*<0.00 and *p* = 0.02 in the 60dB and 70dB noise conditions, respectively), P300 latency (*p*<0.00 in the 70 dB noise condition), and P300 amplitude (*p* = 0.02 in the 60dB noise condition). As can be seen in Fig 2, P1 and P300 latencies were delayed and P300 amplitude smaller in noise conditions compared to the quiet condition. Grand average waveforms to standard and target stimuli for quiet and noise conditions are shown in Figs 3 and 4. Regarding the other variables such as ear and sex, ear effects were observed only for N1 amplitude (*p*<0.00, right ear > left ear) and sex effects only for P1 latency (*p*<0.00, male > female) and P300 amplitude (*p* = 0.02, male < female). Interactions between noise condition and ear were observed for N1 amplitude (*p* = 0.04, right ear < left ear in the condition noise 70dB) and P300 latency (*p* = 0.04, right ear > left ear in the condition noise 60 dB). Age effects on P300 were observed from age 11 onwards, with earlier mean latencies (p = 0.02 at age 11 and p<0.00 at ages 12 and 13) and smaller mean amplitude at age 11 (p<0.00) and larger amplitude at age 13 (p<0.00). As can be seen in Fig 4, P300 latency changes across conditions seem to be more evident from age 11 while P300 amplitude changes seem to appear as early as 8 years old, though no statistically significant changes were found before age 11. Parameter estimate tables can be found in (**S1 Appendix**).

### Experiment 2

**Fig 5:**
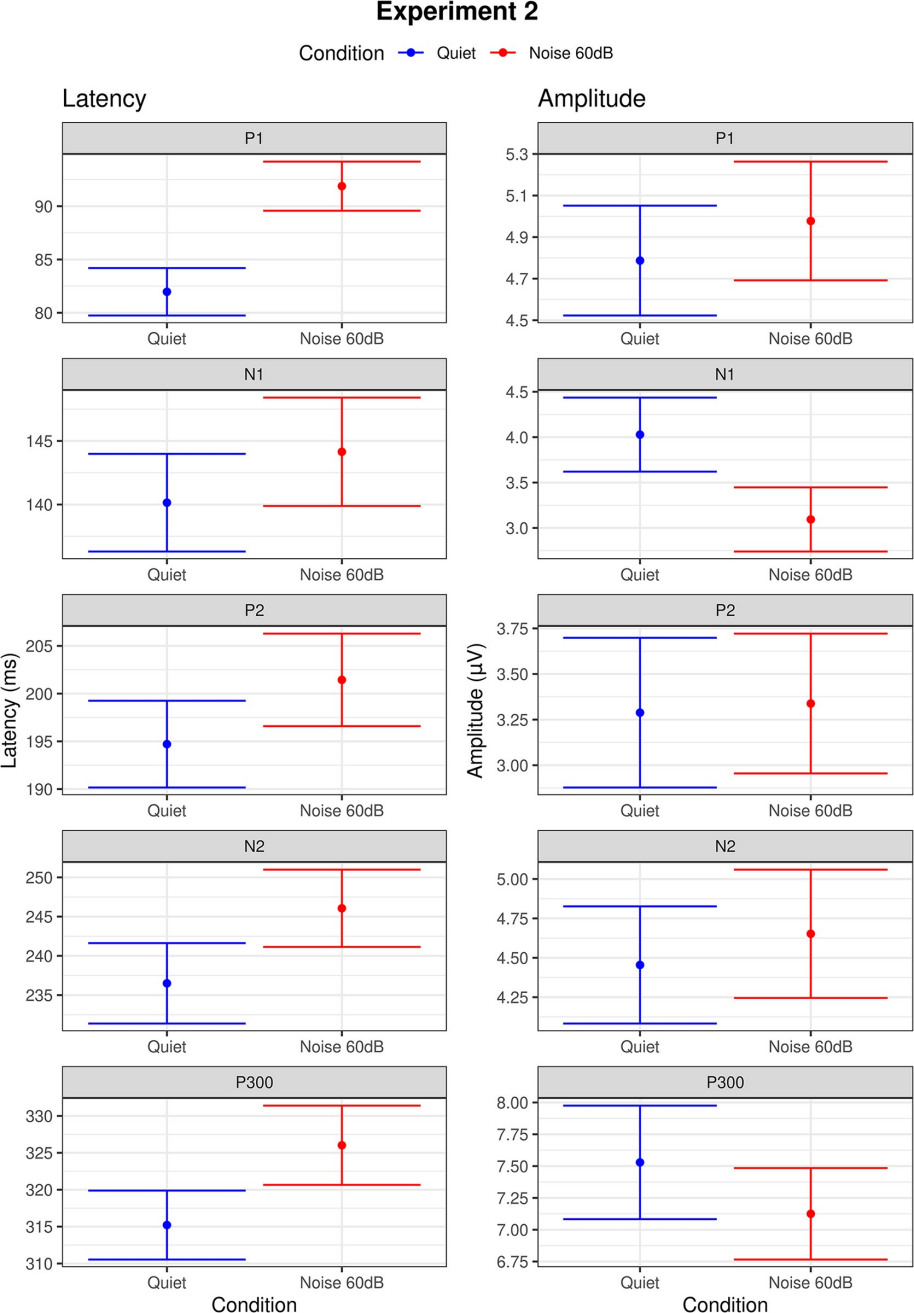
Error bars showing the means and standard errors for the latencies (ms) and amplitudes (μV) of CAEPs obtained with speech stimuli in the conditions quiet (in blue) and noise 60 dB (in red). Dots indicate the mean and error bars indicate the margin of 1 standard deviation around the mean.

**Fig 6:**
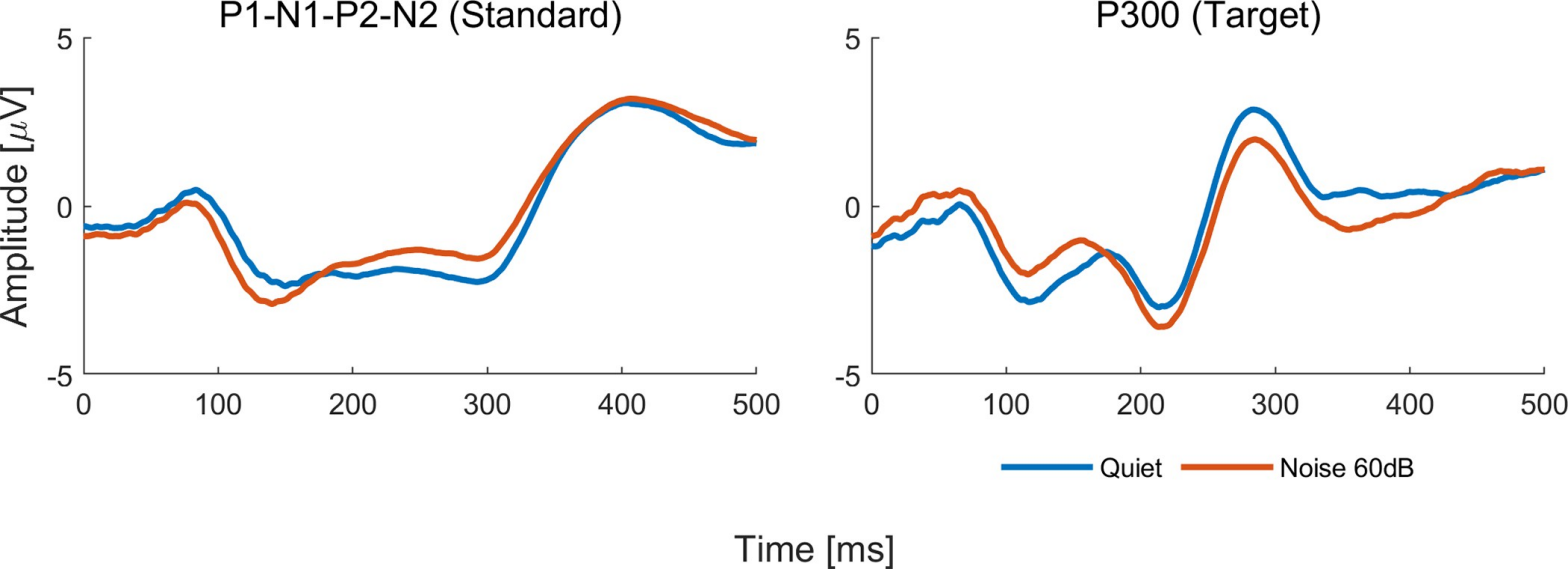
Grand average waveforms for responses to speech stimuli in quiet (blue line) and in competing contralateral noise at 60 dB (red line).

**Fig 7:**
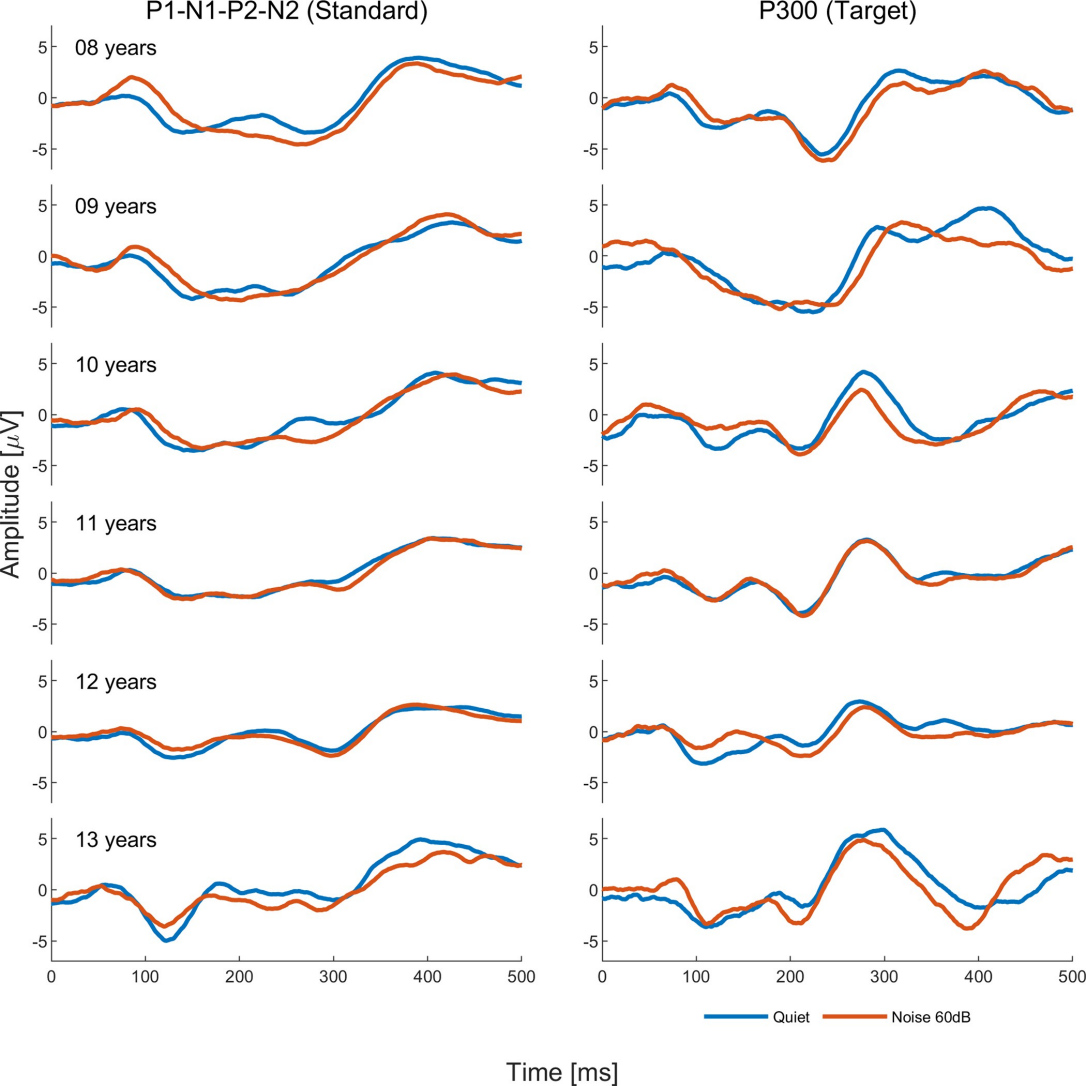
Grand average waveforms by age for responses to speech stimuli in quiet (blue line) and in competing contralateral noise at 60 dB (red line).

In experiment 2, we used speech stimuli to assess CAEPs in quiet and in the 60 dB contralateral white noise condition. Model analysis showed longer latencies for P1 (p<0.00), P2 (p = 0.03), N2 (p = 0.01), and P300 (p = 0.04) and smaller amplitude for N1 (p = 0.04) in the noise condition compared to quiet, as can be seen in Fig 5. Grand average waveforms to standard and target stimuli for quiet and 60 dB noise conditions are shown in Figs 6 and 7. Ear effects were observed for N1 (p = 0.03), P2 (p = 0.01), and N2 (p = 0.02) latencies with longer latencies in the right ear. Sex effects were found for P1 latency (p = 0.02), N1 latency (p<0.00), P2 latency (p = 0.01), and N2 amplitude (p<0.00) with males presenting longer latencies and smaller amplitude compared to females. No significant interactions were found between noise condition and ear and age effects were observed, in general, for most CAEP components regarding the latency and amplitude measures as can be seen in (**S1 Appendix A**).

We also compared tone burst and speech stimuli in the conditions quiet and 60 dB contralateral white noise using the Kruskall-Wallis test to test for stimuli differences. Results showed significant differences in quiet for P1 latency (RE, p = 0.007) and amplitude (RE, p = 0.018; LE, p = 0.009), N1 latency (RE, p<0.001; LE, p<0.001), P2 latency (RE, p<0.001; LE, p<0.001) and amplitude (RE, p<0.001; LE, p = 0.003), and N2 amplitude (RE, p = 0.006; LE, p = 0.014). In the contralateral noise condition, significant stimulus differences were found for P1 latency (RE, p = 0.001; LE, p = 0.02) and amplitude (RE, p = 0.02; LE, p = 0.01), N1 latency (RE, p<0.001; LE, p<0.001), P2 latency (RE, p<0.001; LE, p<0.001) and amplitude (RE, p<0.001; LE, p = 0.004), and N2 latency (RE, p = 0.003; LE, p = 0.008) and amplitude (RE, p = 0.001). Table 2 shows mean latency and amplitude for CAEPs obtained with speech and tone burst stimuli in the conditions quiet and noise 60 dB.

**Table 2:** CAEP latencies and amplitudes obtained with speech and tone burst stimuli in the conditions quiet and noise.

Speech x Tone	Quiet Condition				Noise (60 dB) Condition			
	Tone	Speech	diff	p	Tone	Speech	diff	p
P1—Right Ear (ms)	74.81	84.06	-9.24	**0.007**	78.10	91.84	-13.74	**0.001**
P1—Left Ear (ms)	76.81	79.87	-3.06	0.460	83.57	91.90	-8.33	**0.021**
P1—Right Ear (μV)	3.80	4.79	-0.99	**0.018**	3.86	4.73	-0.86	**0.025**
P1—Left Ear (μV)	3.59	4.78	-1.18	**0.009**	3.81	5.22	-1.41	**0.011**
N1—Right Ear (ms)	108.17	145.25	-37.07	**<0.001**	113.86	145.41	-31.55	**<0.001**
N1—Left Ear (ms)	110.91	135.04	-24.13	**<0.001**	111.84	142.875	-31.03	**<0.001**
N1—Right Ear (μV)	3.96	4.25	-0.29	0.532	3.83	3.15	0.68	0.351
N1—Left Ear (μV)	3.05	3.8	-0.74	0.371	2.83	3.03	-0.19	0.924
P2—Right Ear (ms)	159.15	202.62	-43.47	**<0.001**	162.45	202.62	-40.16	**<0.001**
P2—Left Ear (ms)	153.93	186.79	-32.85	**<0.001**	155.17	200.25	-45.07	**<0.001**
P2—Right Ear (μV)	6.29	3.17	3.11	**<0.001**	6.55	3.64	2.91	**<0.001**
P2—Left Ear (μV)	5.70	3.39	2.30	**0.003**	5.50	3.02	2.47	**0.004**
N2—Right Ear (ms)	227.79	243.38	-15.58	0.201	226.00	248.23	-22.23	**0.003**
N2—Left Ear (ms)	221.79	229.61	-7.82	0.755	219.06	243.88	-24.81	**0.008**
N2—Right Ear (μV)	6.98	4.51	2.46	**0.006**	6.88	4.47	2.40	**0.001**
N2—Left Ear (μV)	6.29	4.39	1.89	**0.014**	5.81	4.82	0.98	0.269
P3000—Right Ear (ms)	322.24	312.37	9.87	0.257	342.21	323.775	18.43	0.055
P300—Left Ear (ms)	323.40	318.05	5.35	0.604	328.85	328.27	0.57	0.941
P300—Right Ear (μV)	7.87	7.55	0.31	0.383	6.65	7.34	-0.69	0.207
P300—Left Ear (μV)	8.03	7.5	0.53	0.541	6.92	6.90	0.02	0.856

Kruskall-Wallis test; Statistical significance level at 5% (p<0.05).

## Discussion

### Experiment 1—Effects of noise on tone burst evoked CAEPs

In experiment 1, we compared the effects of 60 and 70 dB contralateral noise levels on CAEPs obtained with tone bursts. We hypothesized that the 70 dB noise condition would induce greater effects on CAEP responses because it represents a more difficult listening condition [30]. Our results demonstrated an increase in P300 peak latency in the noise 70 dB condition, but not in the noise 60 dB, and a decrease in P300 amplitude in the noise 60 dB, but not in the 70 dB condition. Accordingly, previous studies in children and adults that used monaural tone burst stimuli also showed increased P300 latency in conditions with noise levels that created a signal-to-noise ratio (SNR) of 0 dB [16,21], but not in the conditions with +10 dB SNR [19]. Salisbury et al. [17], on the other hand, found a significant increase in P300 latency despite using a more favorable signal-to-noise ratio (+27 SPL). However, unlike those studies [16,19,21], Salisbury et al. used binaural stimulation, which can enhance CAEP responses both in quiet and noise conditions [24]. Our results suggest that the 60 dB contralateral noise was sufficient to affect (amplitude reduction) the cognitive potential P300 obtained with tone bursts in our sample of children, but did not delay P300 latency as we expected. The presence of significant P300 latency delay only in the 70 dB noise condition may indicate that a more difficult listening condition was necessary to activate the top-down mechanisms when CAEPs were elicited with tone bursts.

As far as we know, there are no studies in the literature that investigated the effects of noise on the P1-N1-P2-N2 obtained with tone bursts in school-aged children. In adult populations, Salo et al. [18] reported N1 amplitude reduction and P2 amplitude increase in normal-hearing adults in the presence of noise. Schochat et al. [19], on the other hand, found a reduction to both N1 and P2 amplitudes. The lack of significant effects of noise on N1-P2-N2 components in our study could be related to the maturational aspects of the CAEPs [31]. In the present research, some children presented morphological immaturity of the waveforms (absent N1 and P2 peaks) [31] in the quiet condition (afferent pathway), which may have “masked” the effects of noise on N1-P2-N2 responses in the condition with noise. Furthermore, children may also present immature corticofugal system (that is, the efferent pathway), which is in accordance with previous studies that suggested that children perform poorly than adults in speech-in-noise perception due to immaturity in the top-down mechanisms [32]. Another possible explanation, however, is that the methodological choices in our study, such as the employed 1 Hz highpass filter, may have contributed to the absence of significant noise effects in experiment 1. There is evidence in the Literature showing that this highpass filter can attenuate and distort ERPs [33–35]. Duncan-Jonson and Donchin [33] demonstrated that as the high-pass filter cutoff became higher, P300 amplitude became correspondingly smaller. Tanner et al. [34] recommended the use of high-pass filter settings between 0.01 and 0.1 Hz for achieving optimal statistical power in detecting true effects while avoiding distortions. Although the principal frequencies of the N1, P2, N2, and P300 components are greater than 3 Hz (implicating that latency shifts and attenuation would be minimal for typical responses) [36], ERP frequencies can be reduced in noise conditions (where increased latencies are expected) and therefore the 1 Hz filter cutoff could be underestimating the differences between quiet and noise conditions. In other words, 1 Hz filtering attenuation is greater in noise versus quiet conditions, which could be “clouding” the differences between conditions. For this reason, we cannot rule out the possibility that the null effects of noise in our study could be due to excessive high pass filtering instead of a maturational characteristic of school-aged children.

### Experiment 2—Effects of noise on speech evoked CAEPs

In experiment 2, we investigated the effect of contralateral noise on CAEP responses to speech stimuli. Our hypothesis was that, because they are complex stimuli and involve more complex neural processing, speech-stimulated CAEPs would be more sensitive than tone bursts to reveal the effects of contralateral noise on sensory and cognitive processing of children. As expected, speech-stimulated CAEPs showed noise induced changes that were not observed in experiment 1 with tone bursts. In experiment 2, all CAEP components (elicited by speech) demonstrated noise induced changes such as N1 amplitude reduction and P1, P2, N2 and P300 latencies delay in the 60 dB contralateral noise condition.

These findings corroborate previous studies in the literature [23,24]. Gustafson et al. [23] described N1, P2 and P3b latency delays for CAEPs elicited by the syllables /da/ and /ga/ in the presence of binaural competitive babble-noise in a sample of normal-hearing aged from 7 to 25 years. Besides the study of Gustafson et al. [23], Morlet et al. [24], described P2 and N2 latency delays (elicited by /da/) in a group of normal developing school-age children when white noise was presented in the ipsilateral and binaural stimulation conditions, but unlike our results those changes were not observed in the contralateral noise condition. The different result in the contralateral noise condition between our study and the one of Morlet et al. [24] may be due to some differences in the parameters used to elicit CAEPs. The literature points out that stimulus elicitor type, competitive noise type, signal-to-noise ratio, and the acquisition paradigm (passive or active listening) can influence CAEP responses both in quiet and noise conditions [30,37,38]. In our study, though P1-N1-P2-N2 peaks were analyzed on the traces corresponding to the standard stimulus (syllable /ba/), children had to pay attention in the task of discriminating and counting the target syllable /da/, whereas in the study by Morlet et al. [24] CAEPs were acquired during a passive listening task (watching a silent movie).

The lack of changes on the amplitude of most CAEPs in our study could be due to the fact that the peak-to-peak measures, used to quantify amplitude, can increase the across-subject variance and reduce statistical power [39]. Peak-to-peak measurements combine the uncertainty of two peaks in every measure, which can confuse changes in amplitude between peaks. To minimize this issue, future studies should measure the fractional area integration (area under the peak), or even the mean amplitude within a fixed width window surrounding each identified peak, which can improve both measurement reliability and specificity, and therefore likely reduce across-subject variability [23,39].

### Experiment 2—Speech *versus* tone evoked CAEPs

Due to stimuli differences on CAEP responses in adult populations [22,37,40], we also sought to verify if there were any differences between CAEPs obtained with each stimulus type (tone burst *versus* speech) in our sample. In general, we found longer P1-N1-P2-N2 latencies, smaller P2 and N2 amplitudes, and greater P1 amplitude in the assessment with speech stimuli when compared to the assessment with tone bursts, which partially corroborate previous studies in adults. Zhang et al. [22] found prolonged MMN, N2b, and P300 for speech (vowels) compared with nonverbal stimuli (complex tones). Contrary to our results, Massa et al. [40] found longer P300 latency and smaller P300 amplitude with speech stimuli compared to tone bursts in normal hearing adults.

In the pediatric population, Martins and Gil [41] and Kileny et al. [42] compared verbal and nonverbal stimuli on CAEPs of cochlear-implanted children and found a trend for longer latencies of P1 [41], N1, P2, N2, MMN and P3a [42], smaller amplitude for P2 and greater amplitude for P3a to speech-stimuli [42], though these differences did not reach statistical significance. To our knowledge, the present study is the first one to report tone burst *versus* speech (syllables) comparisons for P3b in normal hearing school-aged children, but unlike adult studies no statistically significant changes were found between tone and speech P3b in our data.

Given that speech evoked CAEPs are gaining increased interest due to their potential clinical applications [43], our results point to the need for more studies in the normal hearing and developing school-age children to establish normative data for the assessment of the cortical potentials to speech stimuli in clinical settings.

### General discussion, limitations of the study and clinical applications

In the current study, it was possible to observe the effects of contralateral white noise on the CAEPs obtained with tone bursts and speech stimuli. P300 latency delay indicates a slowing in the stimuli evaluation processing while amplitude reduction suggests that fewer neurons were activated in the noise condition, which may be attributed to the inhibitory effect of the efferent system [5,10]. Several researchers have argued that contralateral noise stimulation can activate the olivocochlear bundle, which reduces cochlear gain [5,19,44–46] and therefore decreases the number of afferent primary neurons firing [47], causing a delay in signal transmission along the entire ascending pathway [17,48]. Furthermore, it is known that the efferent system can also modulate auditory afference through descending projections originated in the auditory cortex [8]. Although P300 is an attention-related endogenous potential [29,49], it is not well understood how higher structures of the efferent auditory pathway are activated by noise, causing increased P3b latency as observed in the present study. Nevertheless, our results corroborate previous P300 studies and suggest that the auditory efferent system may be involved, either via the cortico-olivocochlear pathways or the olivocochlear tracts [16,17,20,23].

According to the Literature, this inhibitory effect is important for ruling out competitive noise and therefore facilitating the processing of target sounds in challenging listening environments [8]. Anderson et al. [50] reported noise-induced P1 reduction in both a group of good and a group of poor speech-in-noise school-aged listeners in addition to N2 enhancement only in the group of poor listeners with no changes in the group of good listeners. The authors suggest that the neural coding of the acoustic features of speech may be degraded by noise (as reflected by P1 amplitude reduction in both groups) and that the lack of N2 changes in the good listeners group may indicate greater inhibitory control than the poor listeners, as the former may be recruiting fewer neural resources (due to greater neural efficiency) to synthesize those features into a sensory representation of sound [50].

In the present research, the effects of contralateral noise were more evident in the assessment with speech stimuli instead of tone bursts, probably due to the greater acoustic complexity of speech, which can require from the CANS more complex top-down strategies for processing speech in noise than would be required for processing tones in noise. CAEPs to speech stimuli in quiet and noise conditions can contribute to the understanding of typical and atypical development of the mechanisms involved in speech-in-noise listening, as they reflect spectral and temporal characteristics of speech and the changes induced by background noise.

The present study had a few limitations. As discussed above, the lack of significant changes on the N1-P2-N2 complex in experiment 1 and the lack of changes on the amplitude means of P1, P2, N2, and P300 in experiment 2 can be due to the 1 Hz high-pass filtering cutoff employed in our study, which can attenuate and distort CAEPs in noise conditions [33–35]. Another limitation is related to the peak-to-peak amplitude method of analysis, which increases variability between subjects. Future studies should consider setting the high-pass filter between 0.01 and 0.1 Hz and the analysis approach to be based on the fractional area integration [49], which could contribute for achieving optimal statistical power in detecting true effects, while avoiding distortions, and reducing across-subject variability.

Assessing neural encoding of sounds in background noise is extremely important to help elucidate complaints of difficulty understanding speech in noise. However, studies such as this are still scarce in specialized Literature, especially in school aged children. Establishing the most effective acquisition and recording parameters such as stimulus type, competitive noise type (white noise and speech or babble noise) and noise level, the mode of stimulation (binaural, ipsilateral or contralateral), the acquisition paradigm (passive or active listening), the cutoff filtering, and the method of analysis (*e*.*g*., peak-to-peak, baseline referenced or fractional area) are crucial to promote replicable studies that could contribute to shorten the bridge between research and clinical applications, providing the means for future implementation of assessment protocols that could be feasible to be used in clinical settings and help to improve accuracy in differential diagnosis of the listening difficulties in school-aged children.

## Conclusion

From the analysis of the results, it was possible to verify the effects of noise stimulation on all CAEP components elicited by speech, but the same was not observed in the experiment with tones (that showed changes only in the P1 and P300), which can suggest that speech stimuli would be preferable for investigating the effects of noise on the efferent system using cortical evoked potentials. Therefore, we suggest that the study of noise effects on CAEPs can provide electrophysiological evidence on how difficult listening situations affect sound discrimination and stimulus evaluation at thalamocortical regions.

## Supporting information

S1 Appendix Model parameter estimate tables. (DOCX)

S1 Dataset Dataset underlying the findings. (XLSX)
