# Supplementary material for: Effects of stimuli and contralateral noise levels on auditory cortical potentials recorded in school-age children
Source: PLoS One. 2025 Jan 22;20(1):e0317661. doi: 10.1371/journal.pone.0317661 (PMC11753713; doi:10.1371/journal.pone.0317661)
Supplement: S1 Appendix — (DOCX) [file pone.0317661.s001.docx]

**APPENDIX A**

**Table A.1**

Model parameter estimate table for P1 latency and amplitude means elicited by tone bursts.

| TONE BURST | P1 latency | | | | P1 amplitude | | | |
| --- | --- | --- | --- | --- | --- | --- | --- | --- |
|  | Estimate | Std. Error | t value | Pr(>\|t\|) | Estimate | Std. Error | t value | Pr(>\|t\|) |
| (Intercept) | 77.59 | 1.97 | 39.46 | 0.00 | 4.36 | 0.30 | 14.55 | 0.00 |
| noise(60dB) | 6.76 | 2.17 | 3.11 | **0.00** | 0.22 | 0.33 | 0.65 | 0.51 |
| noise(70dB) | 5.10 | 2.17 | 2.35 | **0.02** | 0.25 | 0.33 | 0.74 | 0.46 |
| Ear(right) | -2.00 | 2.17 | -0.92 | 0.36 | 0.21 | 0.33 | 0.62 | 0.53 |
| Gender(male) | 4.31 | 1.32 | 3.26 | **0.00** | -0.22 | 0.20 | -1.11 | 0.27 |
| Age(9 years) | -2.81 | 2.09 | -1.34 | 0.18 | -0.33 | 0.32 | -1.03 | 0.30 |
| Age(10 years) | -5.30 | 1.95 | -2.72 | **0.01** | -0.52 | 0.30 | -1.76 | 0.08 |
| Age(11 years) | -1.17 | 2.00 | -0.58 | 0.56 | -0.97 | 0.30 | -3.19 | **0.00** |
| Age(12 years) | -3.03 | 1.85 | -1.64 | 0.10 | -1.25 | 0.28 | -4.45 | **0.00** |
| Age(13 years) | -9.23 | 3.35 | -2.76 | **0.01** | -1.99 | 0.51 | -3.91 | **0.00** |
| Noise(60dB):Ear(right) | -3.47 | 3.07 | -1.13 | 0.26 | -0.15 | 0.47 | -0.32 | 0.75 |
| Noise(70dB):Ear(right) | 0.67 | 3.07 | 0.22 | 0.83 | -0.89 | 0.47 | -1.90 | 0.06 |

**Table A.2**

Model parameter estimate table for N1 latency and amplitude means elicited by tone bursts.

| TONE BURST | N1 latency | | | | N1 amplitude | | | |
| --- | --- | --- | --- | --- | --- | --- | --- | --- |
|  | Estimate | Std. Error | t value | Pr(>\|t\|) | Estimate | Std. Error | t value | Pr(>\|t\|) |
| (Intercept) | 104.14 | 3.38 | 30.82 | 0.00 | 2.80 | 0.28 | 10.07 | 0.00 |
| noise(60dB) | 0.93 | 3.40 | 0.27 | 0.78 | -0.22 | 0.28 | -0.77 | 0.44 |
| noise(70dB) | 5.33 | 3.40 | 1.57 | 0.12 | -0.08 | 0.28 | -0.27 | 0.78 |
| Ear(right) | -2.73 | 3.40 | -0.80 | 0.42 | 0.90 | 0.28 | 3.23 | **0.00** |
| Gender(male) | 1.18 | 2.21 | 0.53 | 0.59 | -0.25 | 0.18 | -1.39 | 0.17 |
| Age(9 years) | 9.68 | 3.67 | 2.63 | **0.01** | 0.19 | 0.30 | 0.62 | 0.53 |
| Age(10 years) | -1.44 | 3.43 | -0.42 | 0.67 | 0.49 | 0.28 | 1.74 | 0.08 |
| Age(11 years) | 10.17 | 3.42 | 2.97 | **0.00** | 0.83 | 0.28 | 2.93 | **0.00** |
| Age(12 years) | 8.90 | 3.11 | 2.86 | **0.00** | 0.25 | 0.26 | 0.97 | 0.33 |
| Age(13 years) | 15.53 | 5.30 | 2.93 | **0.00** | 0.04 | 0.44 | 0.08 | 0.94 |
| Noise(60dB):Ear(right) | 4.76 | 4.81 | 0.99 | 0.32 | 0.09 | 0.40 | 0.24 | 0.81 |
| Noise(70dB):Ear(right) | -4.64 | 4.81 | -0.97 | 0.34 | -0.84 | 0.40 | -2.11 | **0.04** |

**Table A.3**

Model parameter estimate table for P2 latency and amplitude means elicited by tone bursts.

| TONE BURST | P2 latency | | | | P2 amplitude | | | |
| --- | --- | --- | --- | --- | --- | --- | --- | --- |
|  | Estimate | Std. Error | t value | Pr(>\|t\|) | Estimate | Std. Error | t value | Pr(>\|t\|) |
| (Intercept) | 143.58 | 3.87 | 37.11 | 0.00 | 7.09 | 0.34 | 21.06 | 0.00 |
| noise(60dB) | 1.24 | 3.87 | 0.32 | 0.75 | -0.20 | 0.34 | -0.59 | 0.55 |
| noise(70dB) | 5.15 | 3.87 | 1.33 | 0.18 | -0.18 | 0.34 | -0.54 | 0.59 |
| Ear(right) | 5.22 | 3.87 | 1.35 | 0.18 | 0.59 | 0.34 | 1.75 | 0.08 |
| Gender(male) | -4.15 | 2.54 | -1.63 | 0.10 | -0.10 | 0.22 | -0.44 | 0.66 |
| Age(9 years) | 15.49 | 4.23 | 3.67 | **0.00** | -0.19 | 0.37 | -0.52 | 0.60 |
| Age(10 years) | 6.56 | 3.84 | 1.71 | 0.09 | -1.16 | 0.33 | -3.48 | **0.00** |
| Age(11 years) | 19.59 | 3.94 | 4.97 | **0.00** | -1.24 | 0.34 | -3.62 | **0.00** |
| Age(12 years) | 15.02 | 3.58 | 4.20 | **0.00** | -2.60 | 0.31 | -8.34 | **0.00** |
| Age(13 years) | 11.74 | 6.10 | 1.92 | 0.06 | -2.65 | 0.53 | -5.00 | **0.00** |
| Noise(60dB):Ear(right) | 2.07 | 5.47 | 0.38 | 0.71 | 0.47 | 0.48 | 0.98 | 0.33 |
| Noise(70dB):Ear(right) | -9.43 | 5.47 | -1.72 | 0.09 | 0.08 | 0.48 | 0.18 | 0.86 |

**Table A.4**

Model parameter estimate table for N2 latency and amplitude means elicited by tone bursts.

| TONE BURST | N2 latency | | | | N2 amplitude | | | |
| --- | --- | --- | --- | --- | --- | --- | --- | --- |
|  | Estimate | Std. Error | t value | Pr(>\|t\|) | Estimate | Std. Error | t value | Pr(>\|t\|) |
| (Intercept) | 223.53 | 3.00 | 74.41 | 0.00 | 7.81 | 0.34 | 22.99 | 0.00 |
| noise(60dB) | -2.73 | 3.22 | -0.85 | 0.40 | -0.48 | 0.36 | -1.30 | 0.19 |
| noise(70dB) | 1.14 | 3.22 | 0.35 | 0.72 | -0.15 | 0.36 | -0.40 | 0.69 |
| Ear(right) | 6.00 | 3.22 | 1.86 | 0.06 | 0.69 | 0.36 | 1.89 | 0.06 |
| Gender(male) | -1.30 | 2.02 | -0.64 | 0.52 | -0.43 | 0.23 | -1.87 | 0.06 |
| Age(9 years) | 5.45 | 3.26 | 1.67 | 0.10 | 0.34 | 0.37 | 0.91 | 0.36 |
| Age(10 years) | -7.56 | 2.93 | -2.58 | **0.01** | -1.62 | 0.33 | -4.88 | **0.00** |
| Age(11 years) | -3.99 | 3.10 | -1.29 | 0.20 | -1.20 | 0.35 | -3.41 | **0.00** |
| Age(12 years) | 1.76 | 2.80 | 0.63 | 0.53 | -3.02 | 0.32 | -9.57 | **0.00** |
| Age(13 years) | -5.91 | 5.46 | -1.08 | 0.28 | -2.82 | 0.62 | -4.56 | **0.00** |
| Noise(60dB):Ear(right) | 0.93 | 4.55 | 0.20 | 0.84 | 0.37 | 0.52 | 0.73 | 0.47 |
| Noise(70dB):Ear(right) | -6.08 | 4.55 | -1.34 | 0.18 | -0.27 | 0.52 | -0.53 | 0.60 |

**Table A.5**

Model parameter estimate table for P300 latency and amplitude means elicited by tone bursts.

| TONE BURST | P300 latency | | | | P300 amplitude | | | |
| --- | --- | --- | --- | --- | --- | --- | --- | --- |
|  | Estimate | Std. Error | t value | Pr(>\|t\|) | Estimate | Std. Error | t value | Pr(>\|t\|) |
| (Intercept) | 329.71 | 4.60 | 71.70 | 0.00 | 8.47 | 0.44 | 19.42 | 0.00 |
| noise(60dB) | 5.44 | 4.94 | 1.10 | 0.27 | -1.10 | 0.47 | -2.35 | **0.02** |
| noise(70dB) | 14.51 | 4.94 | 2.94 | **0.00** | -0.52 | 0.47 | -1.11 | 0.27 |
| Ear(right) | -1.16 | 4.94 | -0.24 | 0.81 | -0.15 | 0.47 | -0.33 | 0.74 |
| Gender(male) | 4.52 | 3.11 | 1.45 | 0.15 | -0.71 | 0.30 | -2.41 | **0.02** |
| Age(9 years) | -5.70 | 4.86 | -1.17 | 0.24 | 0.11 | 0.46 | 0.25 | 0.81 |
| Age(10 years) | 1.99 | 4.51 | 0.44 | 0.66 | -0.43 | 0.43 | -1.00 | 0.32 |
| Age(11 years) | -11.37 | 4.73 | -2.40 | **0.02** | -1.45 | 0.45 | -3.23 | **0.00** |
| Age(12 years) | -17.42 | 4.33 | -4.02 | **0.00** | 0.23 | 0.41 | 0.56 | 0.58 |
| Age(13 years) | -37.50 | 7.19 | -5.22 | **0.00** | 2.16 | 0.68 | 3.17 | **0.00** |
| Noise(60dB):Ear(right) | 14.52 | 6.99 | 2.08 | **0.04** | -0.12 | 0.66 | -0.19 | 0.85 |
| Noise(70dB):Ear(right) | 1.05 | 6.99 | 0.15 | 0.88 | -0.58 | 0.66 | -0.87 | 0.39 |

**Table A.6**

Model parameter estimate table for P1 latency and amplitude means elicited by speech stimuli.

| SPEECH | P1 latency | | | | P1 amplitude | | | |
| --- | --- | --- | --- | --- | --- | --- | --- | --- |
|  | Estimate | Std. Error | t value | Pr(>\|t\|) | Estimate | Std. Error | t value | Pr(>\|t\|) |
| (Intercept) | 79.83 | 3.26 | 24.47 | 0.00 | 5.60 | 0.44 | 12.73 | 0.00 |
| noise(60dB) | 12.03 | 3.14 | 3.83 | **0.00** | 0.44 | 0.42 | 1.04 | 0.30 |
| Ear(right) | 4.18 | 3.14 | 1.33 | 0.19 | 0.01 | 0.42 | 0.03 | 0.97 |
| Gender(male) | 5.75 | 2.44 | 2.36 | **0.02** | -0.39 | 0.33 | -1.19 | 0.24 |
| Age(9 years) | 11.06 | 4.12 | 2.68 | **0.01** | 0.59 | 0.56 | 1.06 | 0.29 |
| Age(10 years) | -1.12 | 4.00 | -0.28 | 0.78 | 0.02 | 0.54 | 0.04 | 0.97 |
| Age(11 years) | -7.04 | 3.77 | -1.87 | 0.06 | -1.71 | 0.51 | -3.37 | **0.00** |
| Age(12 years) | -6.74 | 3.43 | -1.96 | 0.05 | -1.02 | 0.46 | -2.19 | **0.03** |
| Age(13 years) | -6.88 | 6.90 | -1.00 | 0.32 | -2.18 | 0.93 | -2.35 | **0.02** |
| Noise(60dB):Ear(right) | -4.24 | 4.44 | -0.96 | 0.34 | -0.50 | 0.60 | -0.84 | 0.40 |

**Table A.7**

Model parameter estimate table for N1 latency and amplitude means elicited by speech stimuli.

| SPEECH | N1 latency | | | | N1 amplitude | | | |
| --- | --- | --- | --- | --- | --- | --- | --- | --- |
|  | Estimate | Std. Error | t value | Pr(>\|t\|) | Estimate | Std. Error | t value | Pr(>\|t\|) |
| (Intercept) | 147.22 | 5.01 | 29.40 | 0.00 | 3.65 | 0.39 | 9.33 | 0.00 |
| noise(60dB) | 7.83 | 4.64 | 1.69 | 0.10 | -0.77 | 0.36 | -2.12 | **0.04** |
| Ear(right) | 10.21 | 4.64 | 2.20 | **0.03** | 0.46 | 0.36 | 1.26 | 0.21 |
| Gender(male) | 11.47 | 3.62 | 3.17 | **0.00** | 0.46 | 0.28 | 1.61 | 0.11 |
| Age(9 years) | 23.94 | 6.15 | 3.89 | **0.00** | -1.76 | 0.48 | -3.67 | **0.00** |
| Age(10 years) | -21.18 | 5.76 | -3.68 | **0.00** | -1.27 | 0.45 | -2.83 | **0.01** |
| Age(11 years) | -24.70 | 5.75 | -4.29 | **0.00** | -1.44 | 0.45 | -3.21 | **0.00** |
| Age(12 years) | -36.41 | 5.08 | -7.16 | **0.00** | 2.30 | 0.40 | 5.79 | **0.00** |
| Age(13 years) | -27.07 | 9.04 | -2.99 | **0.00** | 0.12 | 0.71 | 0.17 | 0.87 |
| Noise(60dB):Ear(right) | -7.67 | 6.57 | -1.17 | 0.25 | -0.33 | 0.51 | -0.65 | 0.52 |

**Table A.8**

Model parameter estimate table for P2 latency and amplitude means elicited by speech stimuli.

| SPEECH | P2 latency | | | | P2 amplitude | | | |
| --- | --- | --- | --- | --- | --- | --- | --- | --- |
|  | Estimate | Std. Error | t value | Pr(>\|t\|) | Estimate | Std. Error | t value | Pr(>\|t\|) |
| (Intercept) | 190.09 | 6.65 | 28.59 | 0.00 | 3.89 | 0.41 | 9.53 | 0.00 |
| noise(60dB) | 13.46 | 6.16 | 2.18 | **0.03** | -0.37 | 0.38 | -0.98 | 0.33 |
| Ear(right) | 15.83 | 6.16 | 2.57 | **0.01** | -0.22 | 0.38 | -0.58 | 0.56 |
| Gender(male) | 12.78 | 4.81 | 2.66 | **0.01** | -0.35 | 0.30 | -1.18 | 0.24 |
| Age(9 years) | 18.21 | 8.16 | 2.23 | **0.03** | -0.90 | 0.50 | -1.80 | 0.08 |
| Age(10 years) | -23.95 | 7.64 | -3.13 | **0.00** | -1.44 | 0.47 | -3.07 | **0.00** |
| Age(11 years) | -21.54 | 7.63 | -2.82 | **0.01** | -0.79 | 0.47 | -1.68 | 0.10 |
| Age(12 years) | -6.85 | 6.75 | -1.01 | 0.31 | 0.99 | 0.41 | 2.38 | **0.02** |
| Age(13 years) | -22.87 | 12.00 | -1.91 | 0.06 | -2.39 | 0.74 | -3.24 | **0.00** |
| Noise(60dB):Ear(right) | -13.46 | 8.72 | -1.54 | 0.13 | 0.84 | 0.54 | 1.57 | 0.12 |

**Table A.9**

Model parameter estimate table for N2 latency and amplitude means elicited by speech stimuli.

| SPEECH | N2 latency | | | | N2 amplitude | | | |
| --- | --- | --- | --- | --- | --- | --- | --- | --- |
|  | Estimate | Std. Error | t value | Pr(>\|t\|) | Estimate | Std. Error | t value | Pr(>\|t\|) |
| (Intercept) | 240.53 | 6.02 | 39.95 | 0.00 | 6.36 | 0.44 | 14.59 | 0.00 |
| noise(60dB) | 14.26 | 5.74 | 2.48 | **0.01** | 0.44 | 0.42 | 1.05 | 0.30 |
| Ear(right) | 13.76 | 5.74 | 2.40 | **0.02** | 0.12 | 0.42 | 0.29 | 0.77 |
| Gender(male) | 5.09 | 4.41 | 1.15 | 0.25 | -1.56 | 0.32 | -4.88 | **0.00** |
| Age(9 years) | 10.10 | 7.65 | 1.32 | 0.19 | 0.19 | 0.55 | 0.34 | 0.74 |
| Age(10 years) | -37.68 | 7.78 | -4.84 | **0.00** | -1.16 | 0.56 | -2.07 | **0.04** |
| Age(11 years) | -29.28 | 6.82 | -4.29 | **0.00** | -2.34 | 0.49 | -4.75 | **0.00** |
| Age(12 years) | -8.02 | 6.20 | -1.29 | 0.20 | -1.60 | 0.45 | -3.57 | **0.00** |
| Age(13 years) | -35.94 | 12.81 | -2.81 | **0.01** | -3.83 | 0.93 | -4.14 | **0.00** |
| Noise(60dB):Ear(right) | -9.41 | 8.12 | -1.16 | 0.25 | -0.48 | 0.59 | -0.81 | 0.42 |

**Table A.10**

Model parameter estimate table for P1 latency and amplitude means elicited by speech stimuli.

| SPEECH | P300 latency | | | | P300 amplitude | | | |
| --- | --- | --- | --- | --- | --- | --- | --- | --- |
|  | Estimate | Std. Error | t value | Pr(>\|t\|) | Estimate | Std. Error | t value | Pr(>\|t\|) |
| (Intercept) | 336.50 | 5.49 | 61.27 | 0.00 | 7.35 | 0.58 | 12.77 | 0.00 |
| noise(60dB) | 10.22 | 4.96 | 2.06 | **0.04** | -0.59 | 0.52 | -1.14 | 0.26 |
| Ear(right) | -5.68 | 4.96 | -1.14 | 0.26 | 0.06 | 0.52 | 0.11 | 0.91 |
| Gender(male) | -1.38 | 3.78 | -0.37 | 0.71 | 0.20 | 0.40 | 0.49 | 0.62 |
| Age(9 years) | 7.81 | 6.72 | 1.16 | 0.25 | -0.22 | 0.70 | -0.31 | 0.76 |
| Age(10 years) | -33.73 | 6.92 | -4.88 | **0.00** | 1.51 | 0.73 | 2.08 | **0.04** |
| Age(11 years) | -43.99 | 6.03 | -7.29 | **0.00** | 0.38 | 0.63 | 0.60 | 0.55 |
| Age(12 years) | -5.92 | 5.77 | -1.03 | 0.31 | -0.98 | 0.60 | -1.63 | 0.11 |
| Age(13 years) | -38.31 | 8.07 | -4.75 | **0.00** | 1.38 | 0.85 | 1.63 | 0.11 |
| Noise(60dB):Ear(right) | 1.18 | 7.02 | 0.17 | 0.87 | 0.38 | 0.74 | 0.52 | 0.61 |
